# Sexually Dimorphic Effects of a Western Diet on Brain Mitochondrial Bioenergetics and Neurocognitive Function

**DOI:** 10.3390/nu13124222

**Published:** 2021-11-24

**Authors:** Magen N. Lord, Jun-Won Heo, Albino G. Schifino, Jessica R. Hoffman, Kristen N. Donohue, Jarrod A. Call, Emily E. Noble

**Affiliations:** 1 Department of Nutritional Sciences, University of Georgia, Athens, GA 30602, USA; magen.lord@uga.edu (M.N.L.); jessica.hoffman@uga.edu (J.R.H.); kristen.donohue@uga.edu (K.N.D.); 2 Department of Kinesiology, University of Georgia, Athens, GA 30602, USA; junwon.heo@uga.edu (J.-W.H.); ags27911@uga.edu (A.G.S.); call@uga.edu (J.A.C.); 3 Regenerative Bioscience Center, University of Georgia, Athens, GA 30602, USA

**Keywords:** mitochondrial respiration, metabolic flexibility, hippocampus, learning and memory, obesity

## Abstract

A Western diet (WD), high in sugars and saturated fats, impairs learning and memory function and contributes to weight gain. Mitochondria in the brain provide energy for neurocognitive function and may play a role in body weight regulation. We sought to determine whether a WD alters behavior and metabolic outcomes in male and female rodents through impacting hippocampal and hypothalamic mitochondrial bioenergetics. Results revealed a sexually dimorphic macronutrient preference, where males on the WD consumed a greater percentage of calories from fat/protein and females consumed a greater percentage of calories from a sugar-sweetened beverage. Both males and females on a WD gained body fat and showed impaired glucose tolerance when compared to same-sex controls. Males on a WD demonstrated impaired hippocampal functioning and an elevated tendency toward a high membrane potential in hippocampal mitochondria. Comprehensive bioenergetics analysis of WD effects in the hypothalamus revealed a tissue-specific adaption, where males on the WD oxidized more fat, and females oxidized more fat and carbohydrates at peak energy demand compared to same-sex controls. These results suggest that adult male rats show a susceptibility toward hippocampal dysfunction on a WD, and that hypothalamic mitochondrial bioenergetics are altered by WD in a sex-specific manner.

## 1. Introduction

Named for its origin in the global west, the Western diet (WD) has become popular in eastern and developing nations [1]. The globalization of the WD makes understanding its impact on health of critical importance. High in saturated fat and added sugar, WDs promote cognitive dysfunction, but the mechanisms driving the impairments are not fully understood [2,3,4,5,6,7,8,9,10,11,12,13,14,15,16]. The hippocampus, a brain region associated with both learning and memory function as well as food intake control [17], is highly vulnerable to the deleterious effects of WD intake [5,15,18]. In addition to promoting cognitive dysfunction, WDs have been shown to induce weight gain and obesity [5,18]. Diet-induced obesity results from a failure of homeostatic mechanisms to make appropriate adjustments to energy intake and expenditure when consuming a particular diet. These homeostatic mechanisms have been suggested to primarily exist in the hypothalamus of the brain [19,20]; however, the hippocampus has also been shown to be responsive to interoceptive cues of energy balance and may play an influential role in body weight regulation [21,22,23]. Given the role of these brain regions in regulating body weight, we hypothesized that dietary factors comprising an obesogenic WD will negatively impact cellular function in these regions. Herein, we investigated the impact of a WD on cellular energy metabolism with a focus on mitochondrial bioenergetics in both the hippocampus and hypothalamus of mature rats. Our overall goal was to determine whether long-term consumption of a WD fundamentally impacts mitochondrial bioenergetics in the hippocampus and hypothalamus and if maladjustment of neural mitochondria to a WD might be a contributing factor in diet-induced obesity and cognitive dysfunction.

Many commercially available WDs for animal research contain fat and sugar in set ratios in a single pellet form; however, as Slomp et al. eloquently addressed in a recent review, choice is an important component of the WD and may be one of the more obesogenic characteristics of WD consumption, as is the availability of caloric liquids [24]. Therefore, to better model a human obesogenic diet, we employed a choice diet consisting of pellets containing 60% calories from fat and a caloric liquid consisting of an 11% high fructose corn syrup (HFCS) solution, an amount of HFCS comparable to that in a soda. The choice diet also enabled us to observe whether sex differences occur in macronutrient consumption on a WD. Owing to a historic overrepresentation of males as a study subject in feeding research, there is a paucity of data available on sex differences in macronutrient preferences on an obesogenic diet. When given high-fat food pellets, there is evidence to suggest that sex differences exist in the metabolic effects of diet-induced obesity in rodents [25,26,27] and long-term feeding of high-fat food pellets differentially impacted peripheral mitochondrial adaptations to the diet in rats [28]. In the present study, we utilized a choice WD such that we could determine whether males and females self-select different macronutrients, to determine whether sexually dimorphic effects occur in the impact of the diet on metabolic disease, brain mitochondrial adaptations, and neurocognitive function.

Regardless of sex, cellular energy metabolism is of vital importance to neurocognitive functioning, as the brain relies on functional mitochondria for energy. For example, mitochondrial dysfunction in the brain has been linked to several diseases of the nervous system [29], and slight alterations in brain mitochondrial function impair neurocognitive outcomes [30,31]. In addition to reduced ATP production, disruptions in mitochondrial function can result in an increase in formation of reactive oxygen species (ROS) [32,33], and due to the comparatively limited antioxidant defense system in brain tissues, excessive production of ROS in the brain may lead to neurodegenerative disorders [34]. The activity of the complexes of the mitochondrial electron transport chain is influenced by the mitochondrial membrane potential (∆ψm) and the rate of ROS production [34,35]. Furthermore, these factors can be influenced by the presence of uncoupling proteins in the mitochondrial membrane [36], a channel in the inner mitochondrial membrane permeable to hydrogen ions. Previously, it has been shown that high-fat diets confer altered mitochondrial bioenergetics, but the mechanisms responsible for these alterations is unknown [31]. In the present study, we investigated the impact of a choice WD on cellular energy metabolism via mitochondrial bioenergetics, ROS production, and uncoupling protein levels.

## 2. Materials and Methods

### 2.1. Subjects

Adult male and female Sprague Dawley (9–10 weeks, 20 males weighing ~300 and 24 females ~200 g) were obtained from Envigo (Indianapolis, IN, USA). Upon arrival rats were individually housed in a temperature-controlled vivarium (22 °C) with ad libitum access to water and standard rat chow (LabDiet 5053, LabDiet, St. Louis, MO, USA) and acclimated to a 12 h:12 h reverse light/dark cycle for one week. Male and female rats were housed in separate rooms and scrubs, coats, and gloves were changed between handling males and females. Rats were handled and weighed daily beginning during the acclimation period and throughout the experiment. All procedures were approved by the Institute of Animal Care and Use Committee at the University of Georgia (Athens, GA, USA), the protocol numbers were A2020 06-022-Y2-A4 and A2020 06-012-Y2-A16.

### 2.2. Diet Protocol

Following the acclimation period, experimental diet groups were randomized to either a Western diet group (WD; *n* = 10 males, *n* = 12 females) consisting of ad libitum access to high-fat diet (Research Diets D12492; 60.3% kcal fat, 19.6% kcal sugar, 20.1% kcal protein), a bottle of 11% high fructose corn syrup (HFCS, Type 55; Best Flavors, Orange, CA, USA) solution, and a bottle of water. The control diet (CD; *n* = 10 males, *n* = 12 females) consisted of ad libitum access to standard chow (LabDiet 5053, Picolab Rodent Diet 20; 13.1% kcal fat, 62.4 % kcal carbohydrate, 24.5% kcal protein) and two bottles of water. Group assignments were based on body weight to ensure equal starting weights between the groups. For mitochondrial analysis, only four animals’ tissue were processed per day; therefore, start dates were staggered such that four rats (*n* = 2 WD and *n* = 2 CD) were switched to their diet protocol per day to ensure consistency in the total amount of time subjects were on the diet protocol (6 weeks). Body weight, food, HFCS bottles, and water bottles were weighed daily just prior to the onset of the dark cycle.

### 2.3. Intraperitoneal Glucose Tolerance Test

An intraperitoneal glucose tolerance test (IPGTT) is a reliable measure of glucose clearance from the blood and is used as an indicator of the subjects’ insulin secretion and/or sensitivity [37]. All animals were on their respective diet protocol for approximately 35 days (±2 days) when the IPGTT was conducted. Subjects were fasted overnight (8 h) prior to conducting the test.

Two hours prior to the onset of the dark cycle (i.e., Zeitgeber time (ZT) 10), blood samples were collected via tail snip and baseline blood glucose was measured using a glucometer (One touch Ultra2, LifeScan Inc., Milpitas, CA, USA). Each animal was then intraperitoneally (IP) injected with dextrose solution in 0.9% physiological saline (1.5 g/kg body weight) using a 26-gauge needle and 1 mL syringes. Blood glucose was recorded every 30 minutes post injection over 2 h as previously described [4]. Glucose (mg/dL) was plotted against time (min) and the area under the curve (AUC) was estimated using the trapezoidal method in Prism (version 9.1.2, GraphPad Software Inc., San Diego, CA, USA).

### 2.4. Behavioral Testing and Experimental Timeline

For all behavioral testing, experiments began at ZT 14.5 and time of testing was counterbalanced between groups. Males and females were tested in separate rooms, and rats were transferred from their home cage to a transfer cage containing only bedding and taken to the respective behavior room depending on sex. Distance from housing rooms to behavior rooms was equal between the sexes. All testing equipment was routinely cleaned using 10% ethanol before and after each trial to eliminate olfactory cues from preceding animals. The researchers were obscured using black curtains when the animal was undergoing testing. The timeline of behavioral experiments is as follows: Prior to hippocampal-dependent memory testing, animals were tested for anxiety-like behavior in the elevated plus maze after approximately 37 days (±2 days) on their respective diet protocol. At 6 weeks on the diet protocol, animals were tested in the hippocampal-dependent novel location recognition task. Animals were sacrificed the day following the novel location recognition task.

### 2.5. Anxiety-like Behavior

The elevated plus maze (EPM) was used to test anxiety-like behavior according to the methods of Walf et al. [38], with some modifications. The EPM apparatus is made of treated wood and consists of 2 opposing open arms (8.75 × 45 cm) and 2 opposing closed arms (10.3 × 45 cm) with walls that are 40 cm high. The EPM stands so that the rodents are elevated 48 cm above the floor. Rats are placed in the center of the maze with their heads facing toward the open arm away from the experimenter. An overhead video camera is used to record the behavior of the rat over a 5 min period. A closed arm entry is counted when the rat’s center is registered as being in a closed arm. An open arm entry is recorded when the rat’s center is registered as being in an open arm. Testing was recorded and analyzed using ANYmaze software (Stoelting, CO, USA).

### 2.6. Novel Location Recognition Task

The novel location recognition (NLR) task is a measure of hippocampal-dependent learning and memory and has been described previously [39]. The arena is a 66-quart plastic tote (23.98 in. × 16.81 in. × 13.05 in.) striped on the outside with yellow and white tape on two of the adjacent sides. Subjects are habituated to the empty arena for 4 consecutive days for 10 min per day prior to test day. On test day, the familiarization phase consists of placing the animal in the center of the arena with two identical objects (500 mL Fischer bottles filled with green-dyed water) placed in opposite corners of the arena. Subjects are allowed to familiarize themselves to the objects for 3 min followed by a 5 min interval where they are removed from the arena and placed back into the transfer cage and the arena is cleaned with 10% ethanol to eliminate odors. Subjects are then placed back into the arena with one of the objects placed in its original location and the other moved to the corner on the opposite side of the box (novel location). Rats with fully functioning hippocampi will preferentially explore the object that was moved to the novel location more than the unmoved object (familiar location). Exploratory behavior includes being oriented toward the object while sniffing or touching the object with one or both paws. The discrimination index (DI) is calculated by dividing the time spent exploring the novel location by the total time spent exploring both objects. Testing was recorded using an overhead camera and videos were scored by two researchers blinded to diet condition and the two scores were averaged for each animal.

### 2.7. Tissue Collection

Rats were fasted for three hours prior to sacrifice. One hour prior to sacrifice, rats were IP injected with 1.5 g/kg of dextrose solution dissolved in 0.9% physiological saline using a 26-gauge needle in order to mimic a fed, energetically replete metabolic state, eliminating the variability introduced when rats are fed ad libitum prior to sacrifice. At the time of sacrifice, animals were anesthetized via isoflurane inhalation and decapitated via guillotine. Brains were rapidly dissected, and the hypothalamus and the left half of the hippocampus collected into microcentrifuge tubes containing MIR05 buffer (0.5 mM EGTA, 3 mM MgCl2, 60 mM potassium lactobionate, 20 mM Taurine, 10 mM KH2PO4, 20 mM HEPES, 110 mM sucrose, 1 g/L bovine serum albumin) for mitochondrial bioenergetics analyses. The right half of the hippocampus was flash frozen in isopentane cooled in dry ice and stored at −80 °C for protein analyses. Perirenal, perigonadal, and subcutaneous fat pads from the left side of the body were collected and weighed postmortem. Isomorphic samples of liver tissue were flash frozen on dry ice and stored at −80 °C for quantification of neutral lipid content.

### 2.8. Quantification of Liver Fat

Quantification of liver fat from uniform liver samples was performed using Oil Red O (ORO) staining for neutral lipids. Livers were cryosectioned at 10 µm in a cryostat (Leica) at −15 °C and adhered to Superfrost Plus slides and stored at −80 °C until ORO staining. A solution of 0.5% ORO in propylene glycol was heated to 100 °C, filtered through coarse filter paper while warm, and allowed to stand at room temperature for 24 hr. On the day of staining, ORO stock solution was heated to 60 °C. Slides containing liver sections were brought to room temperature and submerged in cold 10% formalin for 5 min, then rinsed in 3 changes of distilled water and allowed to air dry for 5 min. Next, sections were submerged in 100% propylene glycol for 3.5 min. Sections were then covered with the preheated ORO working solution and incubated for 9 min in a preheated 60 °C oven. Sections were then submerged in 85% propylene glycol for 3.5 min before being rinsed in 2 changes of distilled water. Slides were allowed to dry for 5 min and mounted using 1:1 glycerol in KPBS mounting medium and cover slipped.

To quantify neutral lipid content images were captured at 20x magnification using a Nikon Eclipse 80i light microscope. Tissue lipid accumulation was quantified based on ORO staining droplet density using Image J software [40]. After using the subtract background tool, the RGB image was converted to an RGB stack, and the green layer was used for densitometry analyses. The threshold tool was set such that only the red lipid accumulation droplets were highlighted, and the highlighted area was quantified. Data analyses are based on the sum of the areas of samples from four sections per animal.

### 2.9. Mitochondrial Respiration (JO2) Control

High-resolution respirometry was performed on hippocampus and hypothalamus brain samples using an Oroboros Oxygraph-2K (Oroboros Instruments, Innsburck, Austria). Experiments were performed at 30 °C in MiR05 respiration medium (0.5 mM EGTA, 3 mM MgCl2, 60 mM potassium lactobionate, 20 mM Taurine, 10 mM KH2PO4, 20 mM HEPES, 110 mM sucrose, 1 g/L bovine serum albumin) added with 20 mM Creatine, 20 U/mL CK) with constant stirring at 750 rpm. Following the dissection of hippocampus and hypothalamus, the tissues were weighed (1~1.5 mg) and placed into respiration chambers. Saponin (50 µg/mL) was added to permeabilize the tissues as described previously [41], then a basal oxygen consumption rate (pmol/sec/mg) was recorded, and this was followed by the addition of respiratory substrates (carbohydrates fuels: 4 mM Pyruvate, 1 mM Malate, 20 mM Succinate; or fat fuels: 4 mM Palmitoyl-carnitine, 1mM Malate) and 5 mM ATP to energize the mitochondria. Mitochondrial oxygen consumption was determined using a creatine kinase energetic clamp technique where steady-state oxygen consumption rates are measured while manipulating the free energy of ATP hydrolysis (∆G_ATP_) as previously described [42]. Sequential phosphocreatine (PCr) titrations were made to final concentrations of 1, 2, 4, 8, 16, 32, 64 mM. All high-resolution respirometry steady-state oxygen consumption data (*J*O_2_) were corrected for basal oxygen consumption and then normalized by citrate synthase activity, an indirect measure/estimate of mitochondrial content. Plotting the *J*O_2_ as a function of the calculated ∆G_ATP_ shows a linear relationship in which the slope represents the conductance (or ease of flow) of the respiratory system under specific substrates constraints as described by [42].

### 2.10. Mitochondrial Membrane Potential (∆ψm) and NAD(P)H/NAD(P)+ Redox States Assays

Fluorescent determination of mitochondrial membrane potential (∆ψm) and nicotinamide adenine dinucleotide redox status (NAD(P)H/NAD(P)+) was simultaneously performed at 30 °C in 0.2 mL of assay buffer using a Horiba Spectrofluorometer (FluoroMax Plus-C; Horiba Instruments Inc., Atlanta, GE, USA) as previously described [42]. The ∆ψm was determined via tetramethylrhodamine, methyl ester (TMRM) by taking the fluorescence ratio of the excitation/emission parameters: excitation/emission (572/590 nm)/(551/590 nm). The NAD(P)H/NAD(P)+ reduction states were measured via fluorescence using excitation/emission parameters, 340/450 nm. Both assays were run in MiR05 respiration buffer (0.5 mM EGTA, 3 mM MgCl2, 60 mM potassium lactobionate, 20 mM Taurine, 10 mM KH2PO4, 20 mM HEPES, 110 mM sucrose, 1 g/L bovine serum albumin) added with 20 mM Creatine, 20 U/mL CK, and 0.2 µM TMRM. Following the dissection of hippocampus and hypothalamus, the tissues were weighed (1~1.5 mg) and placed into cuvettes. Saponin (50 µg/mL) was added to permeabilize the tissues as described previously [41], followed by the addition of respiratory substrates (carbohydrates fuels: 4 mM Pyruvate, 1 mM Malate, 20 mM Succinate; or fat fuels: 4 mM Palmitoyl-carnitine, 1mM Malate) and 5 mM ATP to energize the mitochondria. Sequential PCr titrations were made to final concentrations of 1, 2, 4, 8, 16, 32, 64 mM that matched the mitochondrial respiration control approach. Following the PCr titrations, 1 µM FCCP and 4 mM cyanide were added for induction of 100 % reduction states within the NAD(P)H/NAD(P)+ couple. The fluorescence (excitation/emission, 340/450 nm) signal measured in the presence of the tissues without respiratory fuels was used as the 0% reduction status for the NAD(P)H/NAD(P)+ couple. The reduction states percentage of NAD(P)H/NAD(P)+ was calculated for each ΔG_ATP_ using the following formula: % Reduction = (F − F0%)/(F100% − F0%).

### 2.11. Citrate Synthase Activity

Mitochondrial content was estimated through the analysis of citrate synthase enzyme activity as previously described [43,44]. Briefly, ~10 mg of hypothalamus or hippocampus tissue was homogenized in 33 mM phosphate buffer (pH 7.4) at a muscle to buffer ratio of 1:40 using a glass tissue grinder. Homogenate was incubated with 5,5′-dithio-bis(2-nitrobenzoic acid (DTNB, 0.773 mM), acetyl CoA (0.116 mM), and oxaloacetate (0.441 mM) in 100 mM Tris buffer (pH 8.0). Citrate synthase enzyme activity was monitored from the reduction of DTNB over time via measurement of the change in absorbance (Δ OD) at 412 nm over a 3 min reaction time. Enzyme reaction was initiated by the addition of oxaloacetate, with each sample run in triplicates.

### 2.12. Stain-Free Western Blotting

Stain-free technology eliminates the need for a loading control by allowing the researcher to instead normalize to the total protein in the lane [45], which we have done herein. Frozen hippocampi were taken from −80 °C and homogenized in NP40 Cell Lysis Buffer (Invitrogen), protease inhibitor (Sigma), and phenylmethylsulfonyl fluoride (Life Technologies) using a homogenizer (VWR model 200), set on a vertical rotator for 30 min at 4 °C, centrifuged for 7 min at 12000× *g*, and the supernatant was aliquoted and stored at −80 °C until Western blotting. A small aliquot was used to determine total protein content using the Bradford assay [46]. Once total protein content was determined, quantification of proteins of interest was performed via standard SDS-PAGE. We assessed 4-hydroxynonenol (4HNE; 40–150 kDa), uncoupling protein-2 (UCP2; 32 kDa), and the five oxidative phosphorylation proteins (OXPHOS; ranging 20-55 kDa) using either 10% (4HNE) or 12% (UCP2 and OXPHOS) CriterionTM TGX stain-free gels (Biorad). Following electrophoresis and gel activation in the ChemiDoc XRS+ (ChemiDoc; Biorad), proteins were transferred to either nitrocellulose (4HNE) or polyvinylidene difluoride (UCP2 and OXPHOS) membranes using a semi-dry transfer method in the Trans-Blot Turbo Transfer System and Trans Blot Turbo transfer packs. Membranes were then imaged in a ChemiDoc and blocked for 1 h using 5% nonfat dried milk in tris-buffered saline with 0.1% Tween. Membranes were then incubated in primary antibody overnight (~20 h). Primary antibodies used: anti-4HNE (1:200 dilution, abcam ab46545), anti-UCP2 (1:1000 dilution, Cell Signaling Technology #89326), and OXPHOS antibody cocktail (1:1000, abcam ab110413). The following day, membranes were washed, incubated in either anti-rabbit (4HNE and UCP2) or anti-mouse (OXPHOS) HRP-conjugated secondary antibodies (both from Jackson ImmunoResearch), and detected following 5 min incubation in enhanced chemiluminescent substrate (Biorad). Band intensity was quantified using Biorad ImageLab software, and values were normalized to total protein content within the well containing the quantified band.

### 2.13. Statistical Analyses

All statistical analyses were performed using Prism (version 9.1.2). Body weight and total caloric intake analyses were performed using two-way ANOVA followed by Sidak’s multiple comparisons post hoc test. Two values were extrapolated from surrounding measurements for male caloric intake and body weight data sets to fit full two-way ANOVA models. Values were extrapolated by averaging the day before and the day after the day of the missing value. Mitochondrial *J*O_2_, ∆ψm, NAD(P)H/NAD(P) redox state analyses were conducted using two-way ANOVA. All data were required to pass normality (Shapiro–Wilk) and equal variance tests (Brown-Forsythe F test) before proceeding with the two-way repeated-measures ANOVA. Significant interactions were tested with Bonferroni’s post hoc test to find differences between groups. Group main effects are reported where significant interactions were not observed. An α level of 0.05 was used for all analyses and all values are means ± SEM unless otherwise noted. Comparisons of fat mass, liver fat, glucose tolerance, behavioral measures, and levels of 4HNE, UCP2 electron conductance, maximal *J*O_2_ and oxidative phosphorylation enzymes were analyzed with either Student’s t-test or Welch’s t-test, depending on the variance in the data set. Specific statistics for each data set are described in the corresponding figure legend.

## 3. Results

### 3.1. Food Intake, Body Weight, and Sex Differences in Selection of Calories from Saturated Fat and HFCS Beverage

**Figure 1:**
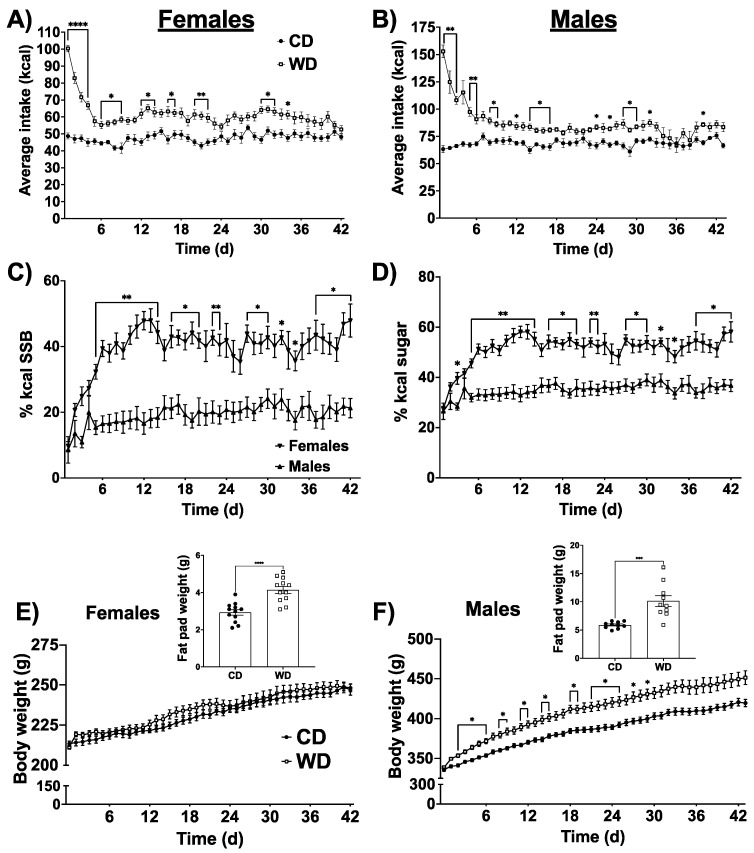
Average total caloric intake for females (time x diet (F(41, 902) = 5.95), *p* < 0.0001) (**A**) and males (time × diet (F(42, 756) = 9.37), *p* < 0.0001) (**B**) consuming either a Western diet (WD) containing 60% fat pellets and an 11% high fructose corn syrup (HFCS) beverage or a chow diet (CD). Percentage of calories consumed from the 11% HFCS (time × sex (F(41, 820) = 2.63), *p* < 0.0001) (**C**). Percentage of total sugar in the diet (from both pellets and 11% HFCS combined) are shown in (**D**; (time x sex (F(41, 820) = 2.63), *p* < 0.0001). Body weight from the day before starting the respective diet to the day of sacrifice is shown for females (**E**) and males (**F**; (F(43, 774) = 4.753, *p* < 0.0001)). Unilateral combined body fat from perirenal, perigonadal, and subcutaneous fat pads at the time of sacrifice is shown for females and males (**E**,**F** inset, respectively). Data are analyzed by two-way repeated-measures ANOVA with Sidak’s post hoc analyses for all figures except for body fat, which are analyzed using Student’s two tailed *t* tests. Data are shown as the means ± SEMs. * *p* < 0.05, ** *p* < 0.01. *** *p* < 0.005, **** *p* < 0.0001.

There was a significant time x diet interaction in both female and male rats, where rats on the WD consumed more calories than rats on the chow diet (*p* < 0.0001 for both sexes, Figure 1A,B). Post hoc analyses revealed that rats in the WD group showed elevated total caloric intake particularly in the early stages of introducing the diet. As subjects in the WD group were given a choice diet, with ad libitum access to both high-fat food pellets (containing 60.3% kcal from fat), and an 11% HFCS sugar-sweetened beverage, we next determined whether there were sex differences in food selection. There was a time × sex interaction, where females consumed significantly more calories from sugar-sweetened beverage compared to males. Females consumed significantly more calories from HFCS beverage than males on a WD with a daily average of ~40% of total kcal coming from HFCS compared with ~19% of kcal from HFCS for males (Figure 1C). As the WD fat pellets also contain sugar, we calculated the total % sugar consumed by males and females on the WD. Females consumed significantly more dietary sugar than males, averaging ~51% of total caloric intake from sugar, while males averaged ~35% of total caloric intake from dietary sugar (Figure 1D). Conversely, males in the WD group consumed significantly more calories from fat, averaging ~49% of daily kcal from fat, while females averaged ~37% (Supplemental Figure S1A). Because males were consuming more fat-rich food pellets than females, and the food pellets contained the only source of dietary protein, males also consumed significantly more protein than females, averaging ~16% of kcal per day from protein, whereas females averaged ~8% of total kcal from protein (Supplemental Figure S1B). Despite elevated total kcal consumption, there were no differences in body weight in females as a result of consuming the WD, though postmortem analyses revealed that the WD caused a greater accumulation of body fat (Figure 1E, *p* < 0.0001). Supplemental Figure S2A–C shows the weights of the individual fat pads weighed from females (perirenal, perigonadal, and subcutaneous fat pads). Conversely, males consuming the WD showed a time x diet interaction for body weight, with animals in the WD group weighing significantly more than animals in the chow group, particularly in the earlier days of consuming the diet. Similar to females, postmortem analyses revealed an elevated accumulation of body fat in the males on the WD (Figure 1D, *p* < 0.0004). Supplemental Figure S2D–F shows the weights of the individual fat pads weighed from males (perirenal, perigonadal, and subcutaneous fat pads).

To investigate whether the increased preference for the sugar-sweetened beverage over the high-fat food pellet are due to circulating reproductive hormones in females, female Sprague Dawley rats (*n* = 5) were ovariectomized and WD intake was measured for 20 days as described above. Ovariectomized (OVX) females on a WD exhibited a pattern near indistinguishable from non-OVX females. Similar to non-OVX females, OVX females showed a reduced % consumption of calories from fat and an increased % kcal from sugar (Supplementary Figure S3A,B). These data suggest that circulating estradiol and progesterone are not contributing to the increased female preference for sugar compared to male rats on a WD.

### 3.2. Consumption of the WD Negatively Impacts Glucose Metabolism and Elevates Liver Fat Content in Males and Females

**Figure 2:**
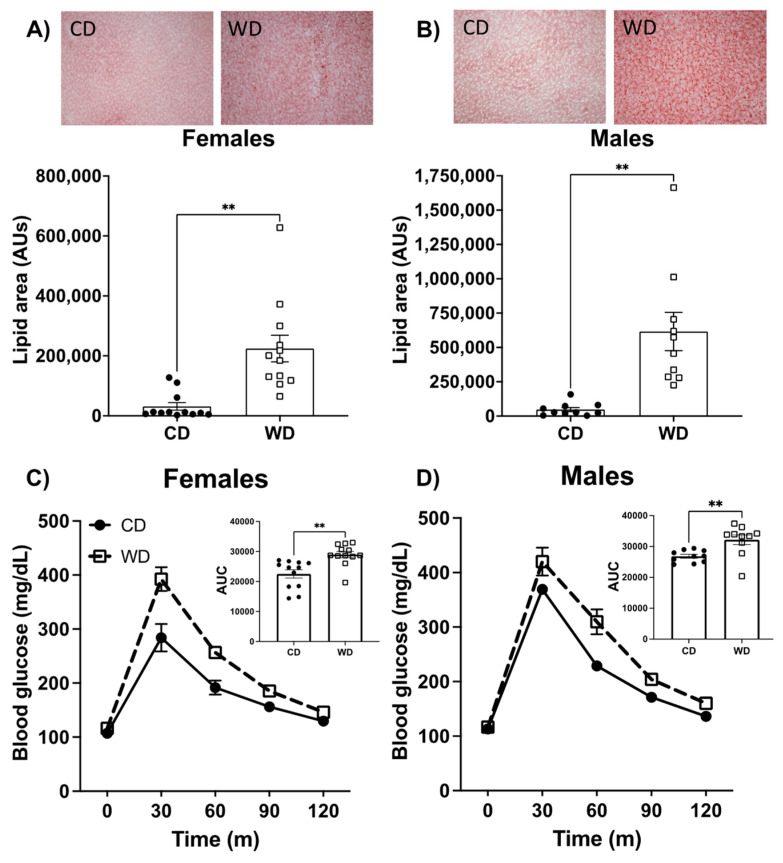
Representative images and quantification of liver fat in females (**A**) and males (**B**) fed either a Western diet (WD) or control diet (CD) using Oil Red O liver stain. Effect of a WD consumption for 5 weeks on blood glucose levels in an intraperitoneal glucose tolerance test in female (**C**) and male (**D**) rats with area under the curve (AUC) shown in each inset. Data are analyzed using a two-tailed Student’s *t* Test ** *p* < 0.01. Data are shown as the means ± SEM. AU= arbitrary units; mg/dL = milligrams/deciliter.

We next examined the impact of the WD on metabolic parameters. Consumption of a WD significantly elevated liver fat in both male and female rats, as determined by Oil Red O staining (Figure 2A,B). Measures of blood glucose metabolism were analyzed using an intraperitoneal glucose tolerance test after approximately 5 weeks on the dietary protocols and before behavioral testing. While there were no differences in fasting blood glucose in either males or females on the diet, the area under the curve was elevated for blood glucose following intraperitoneal injection in both males and females (Figure 2C,D). Taken together, these results show that the WD increased liver fat content and blood glucose clearance similarly in male and female rats.

### 3.3. Consumption of a WD Impairs Hippocampal-Dependent Learning and Memory in Male Rats and Has No Effect on Anxiety-like Behavior

**Figure 3:**
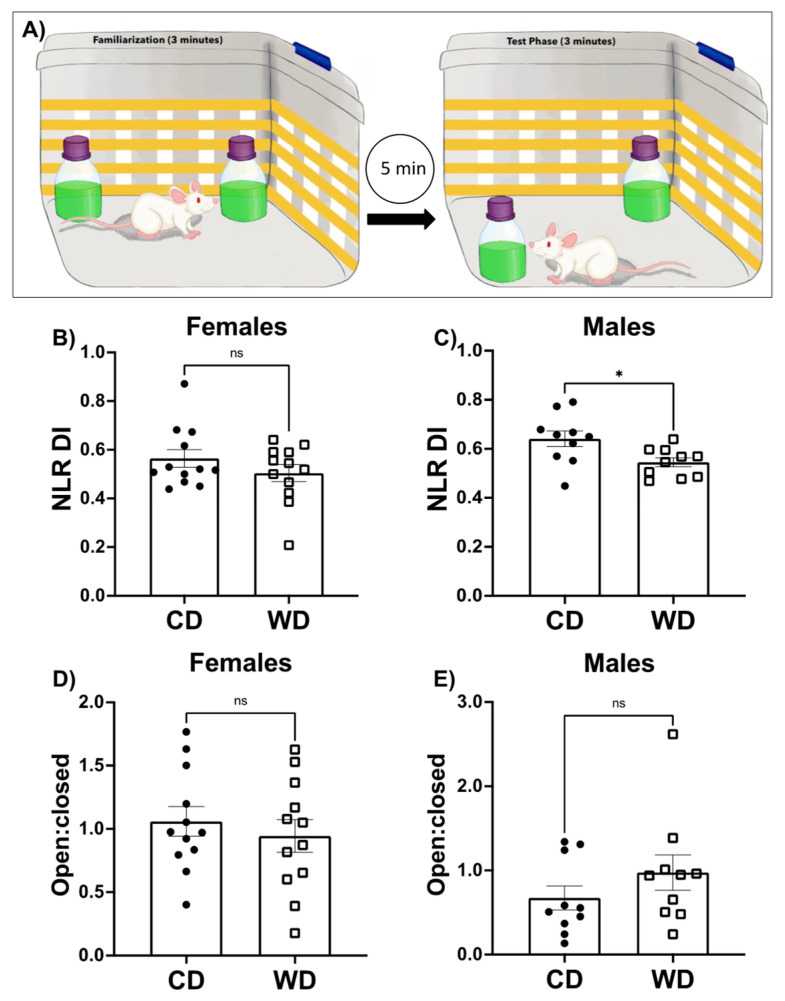
Impact of a Western diet (WD) or chow diet (CD) on performance in neurocognitive tasks. A cartoon representation of the hippocampal-dependent novel location recognition task is shown in (**A**). Following 3 min of familiarization with two identical objects placed in opposite corners of an arena during which time rats equally explore the objects, rats are removed from the arena for 5 min. The rat is then returned to the cleaned arena with the same two objects, but one of the objects is moved to a novel location in the box. Time spent exploring the object in the novel location divided by the total time exploring both objects during the test phase gives a discrimination index score (DI) which is shown for females (**B**) and males (**C**). Results from the elevated plus maze (EPM) are shown for females (**D**) and males (**E**) as the ratio of time spent in the open arm/time spent in the closed arm (open: closed). Data are analyzed using a two-tailed Student’s *t* Test. Data are shown as the means × SEM. * *p* < 0.05.

The novel location recognition (NLR) task was employed to measure hippocampal-dependent learning and memory [39]. A schematic representation of the task is shown in Figure 3A and described in methods. Both CD and WD female groups performed equally well on the NLR task (*p* = 0.2412), though neither of the female diet groups showed a strong preference for the novel location (Figure 3B). Conversely, male rats on CD showed a preference for the novel location, while male rats in the WD group showed impaired discrimination, indicative of impaired hippocampal-dependent spatial memory function (Figure 3C; *p* < 0.05).

WDs have been shown to impact anxiety-like behavior [47]. In the elevated plus maze (EPM), anxiety-like behavior is determined by the amount of time spent in the closed arms of the maze as compared to the open arms of a plus shaped maze that is elevated off the ground. We did not observe differences in anxiety-like behavior in female or male rats on the WD compared to rats fed the CD (Figure 3D,E).

### 3.4. Rationale for Bioenergetics Approach

Standardized mitochondrial oxygen consumption tests at supra-physiological concentrations of ADP can lead to misinterpretations of mitochondrial respiration because mitochondria rarely function in vivo at these extremely low ATP/ADP ratios [48]. Furthermore, mitochondrial energy transduction involves a network of dehydrogenases influencing cellular redox status [e.g., NAD(P)H/NAD(P)], electron transport chain proton pumping and establishment of the proton motive force, and the dissipation of this proton motive force during ATP synthesis based on energy demand (i.e., ATP/ADP ratio) [42,48]. A multi-dimensional diagnostic assessment is therefore required to best assess mitochondrial function under physiological ranges of ATP/ADP ratios. The test described by Fisher-Wellman et al. [42,49] and employed here, uses the enzymatic reaction of creatine kinase (CK) and phosphocreatine (PCr) to stepwise “clamp” ATP/ADP ratios a different ATP free energies such that a high demand for ATP re-synthesis is −12.7 ∆G_ATP_ and a low demand for ATP re-synthesis is −15.0 ∆G_ATP_. As described by Fisher-Wellman et al. [42], when ADP phosphorylation (ATP re-synthesis) is strongly coupled to respiration (i.e., oxygen consumption), then *J*O_2_ is expected to be titrated down proportionately to a rise in ∆G_ATP_. Herein, we simultaneously assessed oxygen consumption rates (*J*O_2_), the proton motive force or mitochondrial membrane potential (∆ψ_m_), and NAD(P)H/NAD(P) redox state during the physiological stress test.

### 3.5. Validation of Mitochondrial Bioenergetics Technique in Brain Tissue

**Figure 4:**
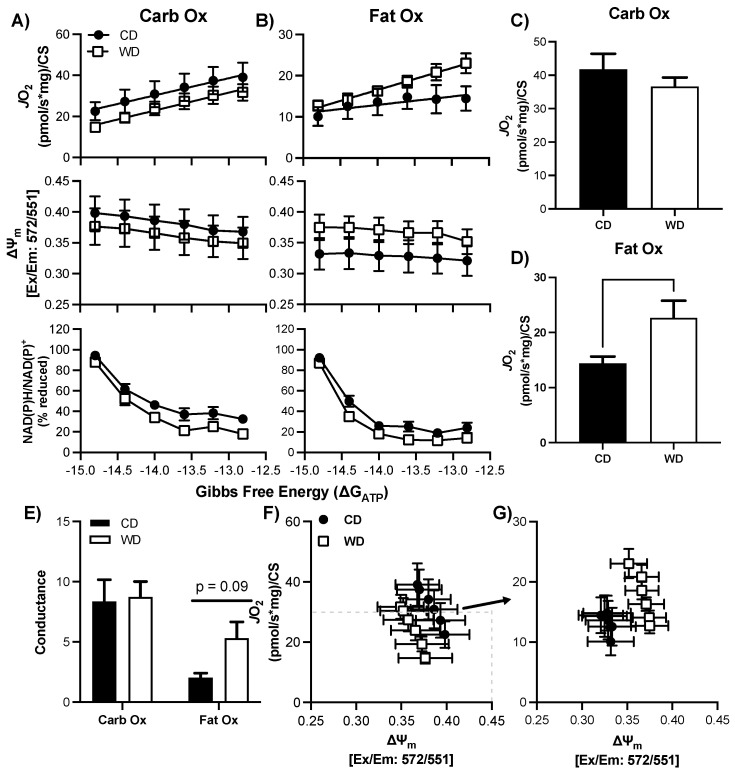
Respiratory control in male hypothalamic mitochondria following consumption of a Western (WD) or chow (CD) diet. **A**–**B**) Changes in mitochondrial respiration (*J*O_2_), membrane potential (∆ψ), and NADH reduction state (NAD(P)H/NAD(P)+) as a function of ATP free energy (∆G_ATP_) and substrate: carbohydrates (**A**) and fats (**B**). (**C**,**D**) Maximal mitochondrial respiration for carbohydrate substrates (**C**) and fat substrates (**D**). (**E**) Conductance slopes calculated from the linear phase of *J*O_2_ as a function of ATP free energy (∆G_ATP_) for carbohydrate and fat substrates. F–G) Relationship between mitochondrial *J*O_2_ and ∆ψ in the presence of carbohydrate (**F**) or fat (**G**) substrates. Differences were analyzed by two-way ANOVA for data shown in panels A and B with factors for diet (CD vs. WD) and ATP free energy. Main effects are noted in the absence of significant interactions (*p* < 0.05). Differences were analyzed by Student’s *t* test for panels (**C**–**E**).

**Figure 5:**
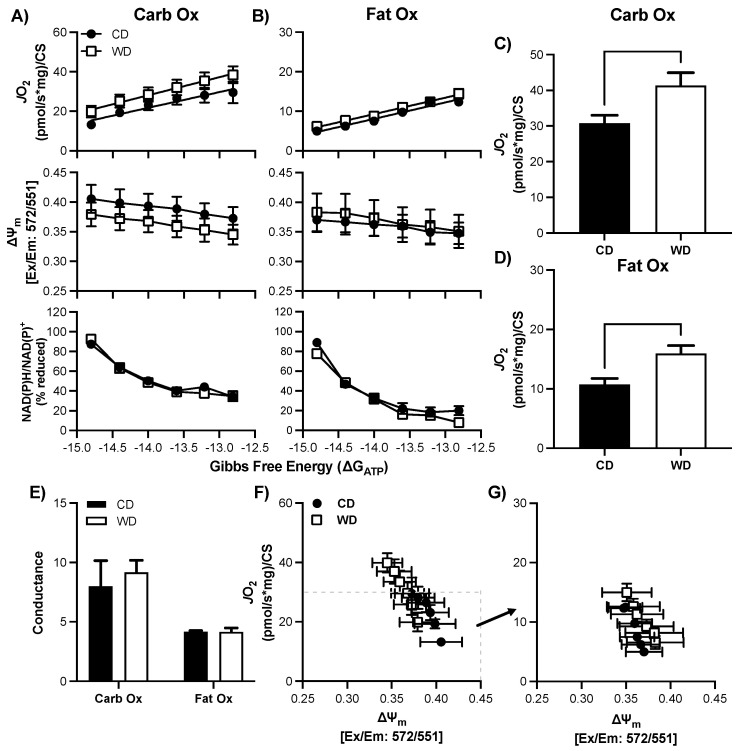
Respiratory control in female hypothalamic mitochondria following consumption of a Western (WD) or chow (CD) diet. (**A**–**B**) Changes in mitochondrial respiration (*J*O_2_), membrane potential (∆ψ), and NADH reduction state (NAD(P)H/NAD(P)+) as a function of ATP free energy (∆G_ATP_) and substrate: carbohydrates (**A**) and fats (**B**). (**C**–**D**) Maximal mitochondrial respiration for carbohydrate substrates (**C**) and fat substrates (**D**). (**E**) Conductance slopes calculated from the linear phase of *J*O_2_ as a function of ATP free energy (∆G_ATP_) for carbohydrate and fat substrates. (**F**–**G**) Relationship between mitochondrial *J*O_2_ and ∆ψ in the presence of carbohydrate (**F**) or fat (**G**) substrates. Differences were analyzed by two-way ANOVA for data shown in panels A and B with factors for diet (CD vs. WD) and ATP free energy. Main effects are noted in the absence of significant interactions (*p* < 0.05). Differences were analyzed by Student’s t test for panels (**C**–**E**).

**Figure 6:**
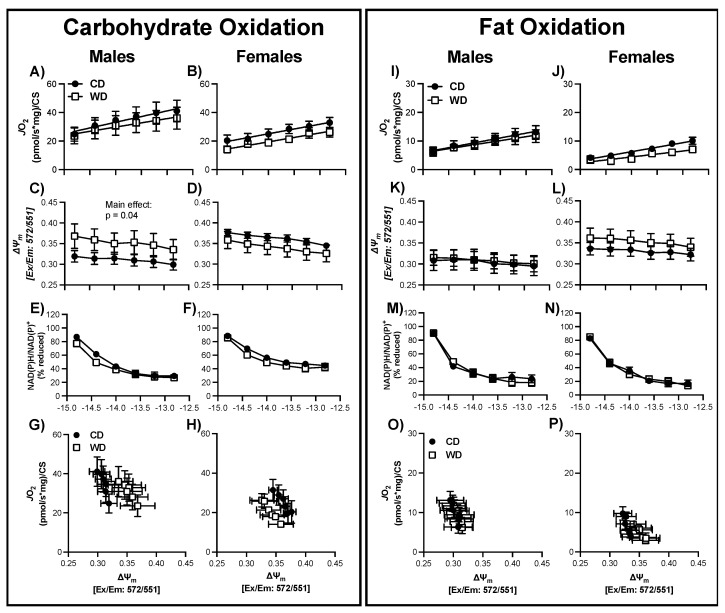
Respiratory control in male and female hippocampal mitochondria following consumption of a Western (WD) or chow (CD) diet. Changes in mitochondrial respiration (*J*O_2_) (**A**,**B**), membrane potential (∆ψ) (**C**,**D**), and NADH reduction state (NAD(P)H/NAD(P)+) (**E**,**F**) as a function of ATP free energy (∆G_ATP_) when carbohydrates are used as a fuel source. Relationship between mitochondrial *J*O_2_ and ∆ψ in the presence of carbohydrate are shown in (**G**,**H**). Changes in mitochondrial respiration (*J*O_2_) (**I**,**J**), membrane potential (∆ψ) (**K**,**L**), and NADH reduction state (NAD(P)H/NAD(P)+) (**M**,**N**) as a function of ATP free energy (∆G_ATP_) when fats are used as a fuel source. Differences were analyzed by two-way ANOVA with factors for diet (CD vs. WD) and ATP free energy. Relationship between mitochondrial *J*O_2_ and ∆ψ in the presence of carbohydrate are shown in (**O**,**P**). Main effects are noted in the absence of significant interactions (*p* < 0.05).

Using the CK-clamp technique, we assessed metabolic flexibility [carbohydrate oxidation (CarbOx) vs. fat oxidation (FatOx)] in the hypothalamus and hippocampus [42]. In both hypothalamus and hippocampus tissue, regardless of sex or substrate (i.e., CarbOx and FatOx), respiratory flux (*J*O_2_) diminished with greater ATP free energies (i.e., more negative ∆G_ATP_, greater ATP/ADP ratio) (Figure 4, Figure 5 and Figure 6). This data agrees with the theoretical and experimental relationship reported between ATP free energies and *J*O_2_ in other tissues [43,50,51]; and thus, establishes the CK-clamp technique as a valid tool for assessment of mitochondrial bioenergetics in brain tissue.

In both hypothalamus and hippocampus tissues, regardless of sex, JO_2_ was greater for CarbOx than FatOx (Figure 4, Figure 5 and Figure 6) implying that pyruvate/malate/succinate are preferred substrates compared to palmitoyl-carnitine/malate. Additionally, the linear phase of the CK-clamp relationship between *J*O_2_ and ∆G_ATP_ has been used previously to define electron conductance (i.e., ease of flow) through the electron transport chain [42,52], and reflects mitochondria sensitivity to changing energetic demands. In support of brain tissue preference for carbohydrate substrates, in general, the conductance was greater for carb fuels compared to fat fuels (e.g., Figure 4E and Figure 5E). In both hypothalamus and hippocampus tissues, dynamic changes in *J*O_2_ were assessed in concert with the NAD(P)H/NAD(P) redox state and mitochondrial membrane potential (∆ψm) to help define how electron potential energy and proton potential energy, respectively, contributes to brain mitochondrial energy production (Figure 4, Figure 5 and Figure 6). In general, the following relationships were consistent regardless of tissue, sex, and substrate: a more negative ∆G_ATP_ produced a greater polarized mitochondrial membrane (i.e., increased ∆ψm) and an increase in the NAD(P)H/NAD(P) redox state. These findings are consistent with a decreased energy demand on the mitochondrial (i.e., greater ATP/ADP ratio), applying a greater backpressure on ATP synthase that increases the proton potential energy and subsequently decreases the pull of electrons through the electron transport chain leading to a more reduced redox state (i.e., less oxidation of NAD(P)H). These bioenergetic relationships agree with similar reports in skeletal muscle, cardiac muscle, brown adipose tissue, kidney, and liver using the CK-clamp technique [43,53,54], and further support the parallel measurement of these systems to comprehensively elucidate brain tissue mitochondria bioenergetics.

### 3.6. Consumption of a WD Alters Hypothalamic Mitochondrial Bioenergetics in Males

There was no statistical difference in basal respiration prior to the CK-clamp procedure between CD and WD for CarbOX (*p* = 0.99, average 11.0 ± 7.7 pmol/sec/mg) or FatOx (*p* = 0.68, average 10.8 ± 6.5 pmol/sec/mg). In the male hypothalamus, there was no significant interaction between ∆G_ATP_ and treatment group, or main effects, for *J*O_2_, ∆ψm, or redox state for either CarbOx or FatOx (Figure 4A,B) through the linear phase of the CK clamp. CarbOx maximal *J*O_2_ was not different between CD and WD (Figure 4C), but maximal *J*O_2_ was 36% greater for FatOx in WD compared to CD (Figure 4D). There was no statistical difference in carb fuel electron conductance between CD and WD, but there was a small trend for greater fat fuel electron conductance in WD (*p* = 0.09, Figure 4E). An intriguing pattern emerges when plotting *J*O_2_ against ∆ψm for CarbOx (Figure 4F) and FatOx (Figure 4G), as there is a prominent upward-right shift for FatOx. The pattern of greater *J*O_2_ and hyper-polarized mitochondrial membrane potential suggests a greater efficiency of oxidative phosphorylation that complements the electron conductance for FatOx (Figure 4E). There was no statistical difference in CS activity between CD and WD (Supplemental Table S1, *p* = 0.51).

### 3.7. Consumption of a WD Alters Hypothalamic Mitochondrial Maximal Respiration in Females

There was no statistical difference in basal respiration prior to the CK-clamp procedure between CD and WD for CarbOX (*p* = 0.50, average 8.7 ± 4.9 pmol/sec/mg) or FatOx (*p* = 0.44, average 9.3 ± 5.4 pmol/sec/mg). Similar to the male hypothalamus, there was no significant interaction between ∆G_ATP_ and treatment group, or main effects, for *J*O_2_, ∆ψm, or redox state for either CarbOx or FatOx (Figure 5A,B) through the linear phase of the CK clamp. Both CarbOx and FatOx maximal *J*O_2_ was greater in WD compared to CD (Figure 5C,D); however, there were no statistical differences between groups in electron conductance (Figure 5E) and no pronounced shifts in the *J*O_2_ ∆ψm relationships (Figure 5F,G). There was no statistical difference in CS activity between CD and WD (Supplemental Table S1, *p* = 0.30).

### 3.8. Consumption of a WD Alters Hippocampal Mitochondrial Membrane Potential in Males during CarbOx

There was no statistical difference in basal respiration prior to the CK-clamp procedure between CD and WD, regardless of sex, for CarbOX (*p* ≥ 0.39, average 10.4 ± 2.7 pmol/sec/mg) or FatOx (*p* ≥ 0.41, average 11.0 ± 4.5 pmol/sec/mg). Regardless of fuel source and sex, there was no significant interaction between ∆G_ATP_ and treatment group for *J*O_2_, ∆ψm, or redox state in hippocampal tissue (Figure 6). For both males and females, there were no statistical differences between CD and WD groups for maximal CarbOx *J*O_2_, maximal FatOx *J*O_2_, and electron conductance (Supplemental Figure S4A–F). There was a main effect of treatment group, independent of ∆G_ATP_, for males with CarbOx substrate indicating a greater, or hyper-polarizing, mitochondrial membrane (Figure 6C). As a result, when plotting *J*O_2_ against ∆ψm there is a right shift such that for any given *J*O_2_ the mitochondrial membrane has a more negative potential (Figure 6G). In contrast to the upward-right shift in the male hypothalamus, hyper-polarization without a corresponding increase in *J*O_2_ can suggest resistance in ATP synthase and creates a cellular environment favorable to electron leak and reactive oxygen species (ROS) production. There was no statistical difference in CS activity between CD and WD, regardless of sex (Supplemental Table S1, *p* ≥ 0.36).

### 3.9. WD Consumption Does Not Alter Uncoupling Protein-2, Oxidative Phosphorylation Enzymes, or Reactive Oxygen Species

Stain-free Western blotting [45] was utilized to quantify levels of uncoupling protein-2 (UCP2), the five oxidative phosphorylation enzymes, and the lipid peroxidation product 4-hydroxynonenal (4HNE), a reactive oxygen species (ROS). No differences were detected between WD and CD animals of either sex in levels of UCP2 (Supplemental Figure S5A,D), 4HNE (Supplemental Figure S5B,E) or complex II, III, IV, or V (Supplemental Figure S5C,F).

## 4. Discussion

It is now well established that a WD promotes obesity and metabolic disease in both rodent and human models, and negatively impacts cognitive functions that rely on the hippocampus, though the mechanisms for these effects are not well understood [5,8,49,55,56]. Our male rodent data similarly show that WD consumption negatively impacts hippocampal function (Figure 3C) and promotes weight and fat gain (Figure 1F), as well as elevated liver fat content (Figure 2B) and impaired glucose tolerance (Figure 2D). Given that the brain is a central orchestrator of body weight and adjusts both energy expenditure and food intake to keep weight stable [50], the obesogenic qualities of a diet must interfere with homeostatic mechanisms in the brain. Therefore, given the critical nature of mitochondria in providing cellular energy to maintain neuronal function and to respond to cellular stress, we further investigated the impact of a WD on mitochondrial bioenergetics in the hippocampus and hypothalamus, two key regions for regulating energy homeostasis.

In contrast to the majority of studies which utilize a single food pellet containing macronutrient ratios that model a WD, our study utilized a choice WD in order to more closely model the human food environment where sugar-sweetened beverages are the largest contributor to total added sugar intake [51]. In our study, added sugar was both a component of the food pellet (which also contained saturated fat and protein) and available via beverage containing 11% HFCS (the amount in commercially available sugar-sweetened beverages). Both males and females showed elevated total calorie intake with the introduction of the WD that declined over time (Figure 1A,B). To our surprise, however, we observed marked sex differences in macronutrient self-selection. Females self-selected more kcal from the sugar-sweetened beverage compared with the males and consumed nearly half of their daily calories from added sugars throughout this study (Figure 1C). Coinciding with the elevated sugar consumption, the female rats reduced their intake of the high-fat food pellet, which also contained dietary protein (Supplementary Figure S1). As female reproductive hormones have been shown to increase the threshold for sweet taste [53], we posited that perhaps circulating female hormones are drivers of sugar preference. Therefore, we investigated whether the sweet preference would be reduced in ovariectomized females, in whom circulating estrogen and progesterone are drastically reduced. We found that ovariectomized females showed similar macronutrient intakes to cycling females (Supplementary Figure S3), and thus estrogen and progesterone are not likely the driving factors for the observed sex differences. Testosterone has been shown to increase fat preference in female rats [54], and thus it is possible that male reproductive hormones drive a fat and/or protein preference in males, but this remains to be determined in a future study. To our knowledge, a similar study design which captures fat vs. sugar preference in male vs. female rats has not been previously reported.

Despite overall greater calorie intake in both males and females, only the males consuming the WD showed overall elevated body weights on the WD (Figure 1). However, both males and females on the WD showed elevated body fat (Figure 1E,F) as well as increased liver fat and an elevated area under the curve in an intraperitoneal glucose tolerance test (Figure 2). Prior research suggests males are more sensitive to negative metabolic consequences of a high-fat diet. For example, compared to females, males on a HFD exhibit elevated fat content in the liver [55], elevated fasting blood glucose, impaired glucose tolerance, and significant weight gain [56]. In our study, both males and females on a WD showed similar levels of metabolic disruption. The females did not show differences in body weight but had elevated percentages of body fat (Figure 1E). Due to their increased consumption of calories from the sugar-sweetened beverage, their protein intake was only approximately 7% of their total calorie consumption (Supplementary Figure S1). It is likely, therefore, that the WD caused a reduced bone and/or muscle mass in the females; however, this prospect was not directly tested in our study.

The hypothalamus plays an important role in modulating energy balance and glucose homeostasis. Additionally, the hypothalamus contains energy sensors that detect ATP production and coordinate/regulate both food intake and energy expenditure to maintain homeostasis [57]. We hypothesized that a central mechanism by which a WD changes the brain is by impacting cellular energy metabolism, more specifically mitochondrial bioenergetics in the hypothalamus. Our data show adaptations in hypothalamic tissue of both male and female rats, though due to dietary choice differences we are unable to determine from these data whether diet per se or biological sex are at the root of these differences. Males on a WD were better able to oxidize long chain fatty acid in hypothalamic tissues (Figure 4D), whereas hypothalamic tissues from females on a WD were better able to oxidize both fats and carbohydrates (Figure 5C,D). These adaptations are quite striking, but not entirely surprising. Evidence suggests that hypothalamic fatty acid oxidation acts as a sensor for nutrient availability and reduces food intake [58,59]. Indeed, inhibiting the transport of these fatty acids via inhibition of the long chain fatty acid transporter CPT1 has been shown to elevate food intake in rodents [59]. In rodents fed a WD, the capacity to oxidize fatty acids in the hypothalamus was enhanced, suggesting an adaptation to the diet which should reduce the orexigenic impact of the diet. Our data reflect that hypercaloric intakes were indeed attenuated in WD fed animals by the time of tissue collection. It would be interesting to determine, in future studies, whether this is not the case immediately following the presentation of the diet, when animals are hyperphagic.

Our results show that elevated consumption of a WD, consisting of saturated fat and added sugar, negatively impacts hippocampal-dependent learning and memory function in male, but not female laboratory rats (Figure 3B,C). There were no differences in anxiety-like behavior, indicating no confounding effects due to anxiety-like behavior in the task (Figure 3D,E). Our findings agree with prior research, showing that a diet high in both saturated fat and added sugar negatively impacts learning and memory function in humans (see [60] for review) and this phenomenon has been extensively shown to occur in adult male rodents [3,5,6,7,8,9,13,14,61,62]. The lack of group differences observed in female rodents has been reported previously [63,64] and may be due to sex differences in the approach to solving a spatial task. Whereas males tend to use environmental cues, a process that relies on the integrity of the hippocampus, females often employ a response strategy that relies on executing a motor response based on sequential cues, a process that does not depend on hippocampal integrity but relies more on the dorsolateral striatum [65,66,67]. Our data show that while there is no effect of a WD on hippocampal-dependent learning and memory function in females, the female rats in the control group did not discriminate which object was in the novel location and the exploration times around the novel object were not above chance. Future studies should focus on alternative methods to test the impact of diet on hippocampal function besides those that rely on spatial navigation strategies, as our data and others suggests that these may not be an appropriate strategy for hippocampal assessment in females.

The hippocampus is involved in body weight regulation by maintaining energy balance as well as learning and memory function. For example, an intact hippocampus is critical for body weight regulation, as damage to the hippocampus promotes excessive food intake due to reduced sensitivity to interoceptive signals of energy states [68,69,70,71,72]. Therefore, hippocampal brain regions might be particularly susceptible to chronic metabolic states with altered macronutrient composition and/or energy intake. Despite extensive evidence that a diet high in fat and sugar impairs hippocampal-dependent learning and memory function, the mechanisms underlying this dysfunction remain unclear. As the cells of the hippocampus have a high energy demand, we set out to determine whether a disruption in mitochondrial bioenergetics might contribute to impaired hippocampal function. Our results indeed show an elevated membrane potential for rate of oxygen consumption in hippocampal tissue of male rats fed a WD (Figure 6). In the physiological state, oxygen is the final receiver of electrons pulled through the electron transport chain (creating two H2O molecules) and this process creates a proton motive force to power ATP production. However, in hyperpolarization states, an electron leak can occur leading to the generation of superoxide radicals [73] and result in tissue damage. For example, long-term consumption (18 weeks) of a high-fat diet has been shown to alter mitochondrial bioenergetics in the cortex of male mice and elevate malondialdehyde, a marker for oxidative stress [31]. Additionally, 32 weeks of high-fat diet-induced obesity led to mitochondrial dysfunction (i.e., decrease in *J*O_2_ and Ca2+ retention capacity, and increase in H_2_O_2_ emission), resulting in impairment of memory function in the hippocampus [74]. Therefore, dysfunctional mitochondria might be a link between diet-induced obesity and brain tissue oxidative damage.

In general, mitochondrial bioenergetics were normal in our 6-week high-fat diet study; however, there were some trends that may allude to what others have reported with long-term super-nutrient exposure. Our data indicates a main effect of WD compared to CD on maximal fat oxidation in both male and female hypothalamus, which indicates a greater ability to produce ATP derived from intermediates of fatty acid oxidation. The increase in mitochondrial respiration indicates possible adaptations that may be a result of increased free fatty acid availability [75]. Interestingly, female hypothalamus demonstrated increased respiration from carbohydrate sources as well. However, when considering membrane polarity as it relates to mitochondrial oxygen consumption (*J*O_2_), WD group exhibits a hyperpolarized state. The hyperpolarized state indirectly indicates ATP synthase backpressure on the oxidative phosphorylation system. Indeed, ATP synthase is a critical mechanism maintaining mitochondrial membrane potential, where dysfunction can result in hyperpolarization and subsequent electron leak/free radical creation.

Oxidative damage is a cumulative outcome whereas oxidative stress is a dynamic relationship between ROS production and antioxidant capacity. Capturing either is difficult and may have eluded our study design. Typically, oxidative damage to membrane lipids (i.e., lipid peroxidation) leads to the formation of highly reactive aldehydes such as 4HNE (assessed herein) and malondialdehyde. In the present study, no differences were observed in levels of 4HNE in males nor females (Supplementary Figure S5B,E). However, oxidative damage can also involve proteins (e.g., protein carbonylation) and DNA (mtDNA adducts). Oxidative stress can be measured by the active production of H_2_O_2_ from respiring mitochondria or via alterations in the antioxidant protein abundance such as the reduced to oxidized ratio of glutathione. Significant portions of the hypothalamus and hippocampus were allocated to mitochondrial bioenergetics and enzyme kinetic assays in this study limiting the capacity of thorough oxidative damage/stress analysis. Future studies evaluating ROS production during mitochondrial respiration with fat and carbohydrate substrates and via Western blot of antioxidant proteins could further elucidate the impact of WD on brain oxidative stress.

## 5. Conclusions

To conclude, our data show sex-specific responses to a WD model where animals self- select from a choice of sugar-sweetened beverage or a high-fat food pellet, where both macronutrient intake and hippocampal and hypothalamic mitochondrial bioenergetics are differentially regulated. In our models, both sexes developed metabolic disease (elevated liver fat, elevated body fat, impaired glucose tolerance) characteristic of diet-induced obesity even though females did not gain weight on the WD. Hippocampal bioenergetics was altered by WD in male rats, who also had accompanying learning and memory deficits. Our data suggest that future research utilizing a more appropriate method to assess hippocampal function in female rats is necessary.

## Data Availability

All data are available upon request.

## Supplementary Materials

The following are available online at https://www.mdpi.com/article/10.3390/nu13124222/s1, Figure S1: Average percentage of kcal from fat and protein in the Western diet group; Figure S2: Body fat accumulation in female and male rats on control diet vs. Western diet; Figure S3: Average intake and macronutrient preferences of ovariectomized females on Western diet; Figure S4: Respiratory control in hippocampal mitochondria following consumption of a Western or control diet; Figure S5: Stain-free Western blotting in the hippocampus of rats fed either a Western or control diet.
