# Supplementary material for: Sexually Dimorphic Effects of a Western Diet on Brain Mitochondrial Bioenergetics and Neurocognitive Function
Source: Nutrients. 2021 Nov 24;13(12):4222. doi: 10.3390/nu13124222 (PMC8705773; doi:10.3390/nu13124222)
Supplement: Supplementary file 1 [file nutrients-13-04222-s001.zip › nutrients-1430662-supplementary.pdf]

Supplemental Methods:

Female ovariectomies:

Once noted that marked macronutrient preferences exist between males and females on a WD (see Figure 1C, D and Supplementary Figure 1), we investigated whether these differences remained following removal of circulating reproductive hormones via ovariectomy of five female Sprague-Dawley rats obtained from Envigo (Indianapolis, IN). Female rats were bilaterally ovariectomized (OVX) via dorsal flank incisions. Bilateral 2 mm incisions were made, ovaries were removed, and fallopian tubes were sutured, as previously described [1,2]. Animals were allowed to recover for one week post-surgery. Following recovery, OVX rats were switched to a Western Diet (WD) consisting of ad libitum access to high fat diet (Research Diets D12492; 60.3% kcal fat, 19.6% kcal sugar, 20.1% kcal protein), a bottle of 11% high fructose corn syrup (HFCS, Type 55; Best Flavors, Orange, CA) solution, and a bottle of water. Body weight, food, HFCS bottles, and water bottles were weighed daily just prior to the onset of the dark cycle for three weeks.

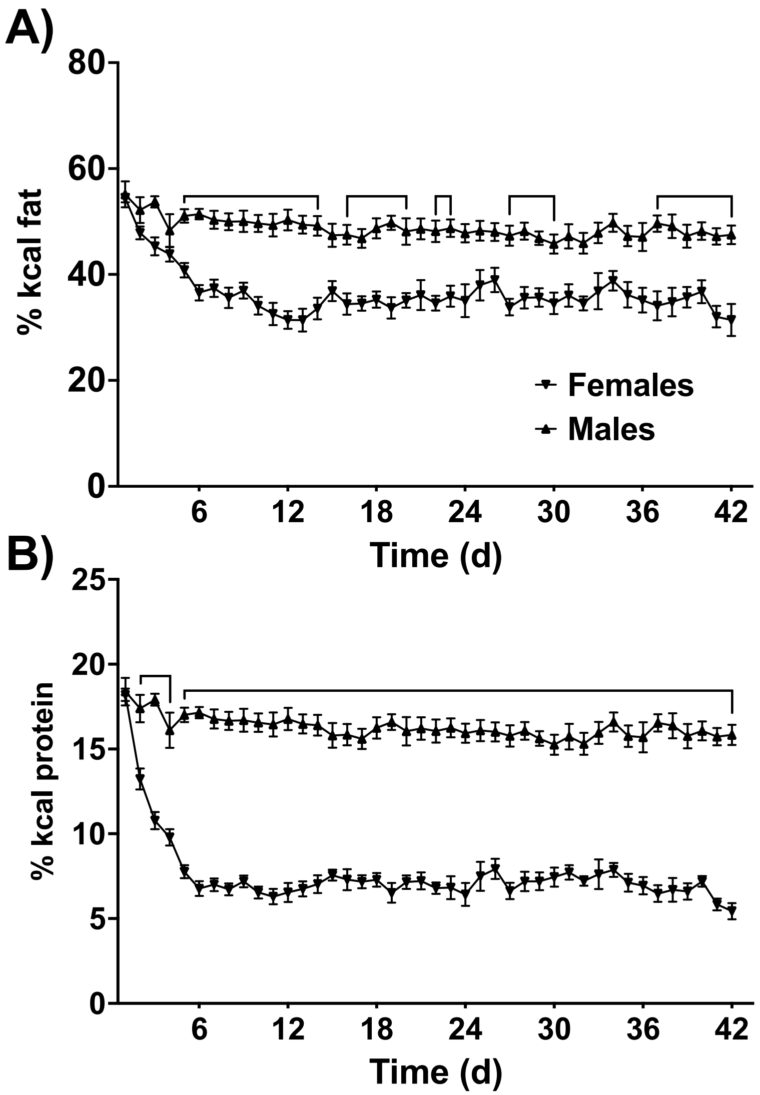

Supplemental Figure S1: Average percentage of kcal from fat and protein in the Western Diet group. Average % kcal from fat intake for females and males (time x diet ($F_{(41, 820)}$ = 2.66), P<0.0001) (A) and average % kcal from protein intake for females and males (time x diet ($F_{(41, 820)}$ = 7.98), P<.0001) consuming a Western Diet (WD) containing 60% fat pellets and an 11% high fructose corn syrup (HFCS) beverage. Data are analyzed by Two-Way Repeated Measures ANOVA with Sidak post hoc analyses. Data are shown as means ± SEMs. *P<0.05, **P<0.01, ****P<0.0001.

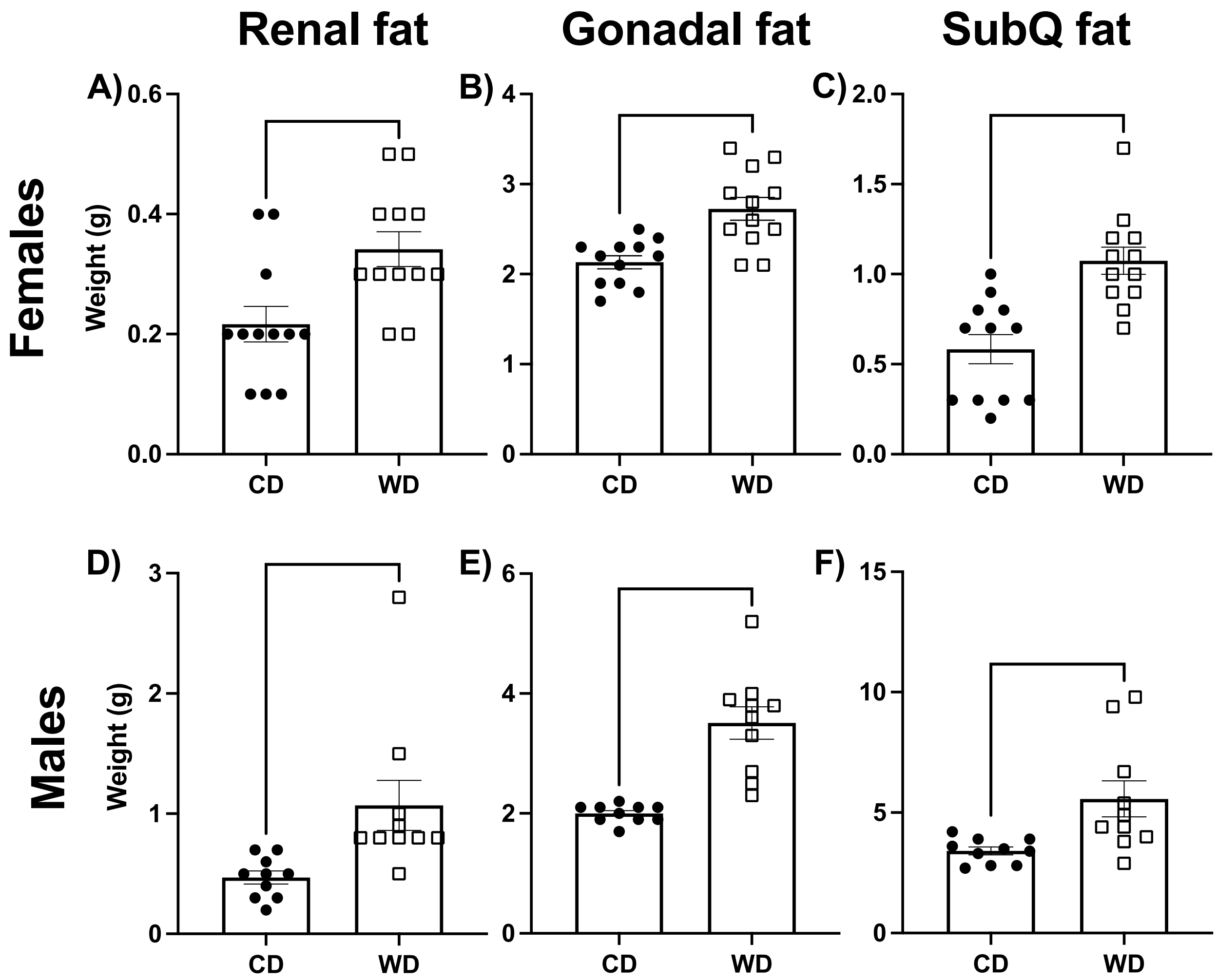

Supplemental Figure S2: Body fat accumulation in female and male rats on control diet vs. Western Diet. Unilateral body fat from perirenal, perigonadal, and subcutaneous fat pads at the time of sacrifice is shown for females (A-C) and males (D-F) consuming either a Western Diet (WD) containing 60% fat pellets and an 11% high fructose corn syrup (HFCS) beverage or a control diet (CD). Data are analyzed using Student's two tailed t tests. Data are shown as means ± SEMs. *P<0.05, **P<0.01, ***P<0.001, ****P<0.0001.

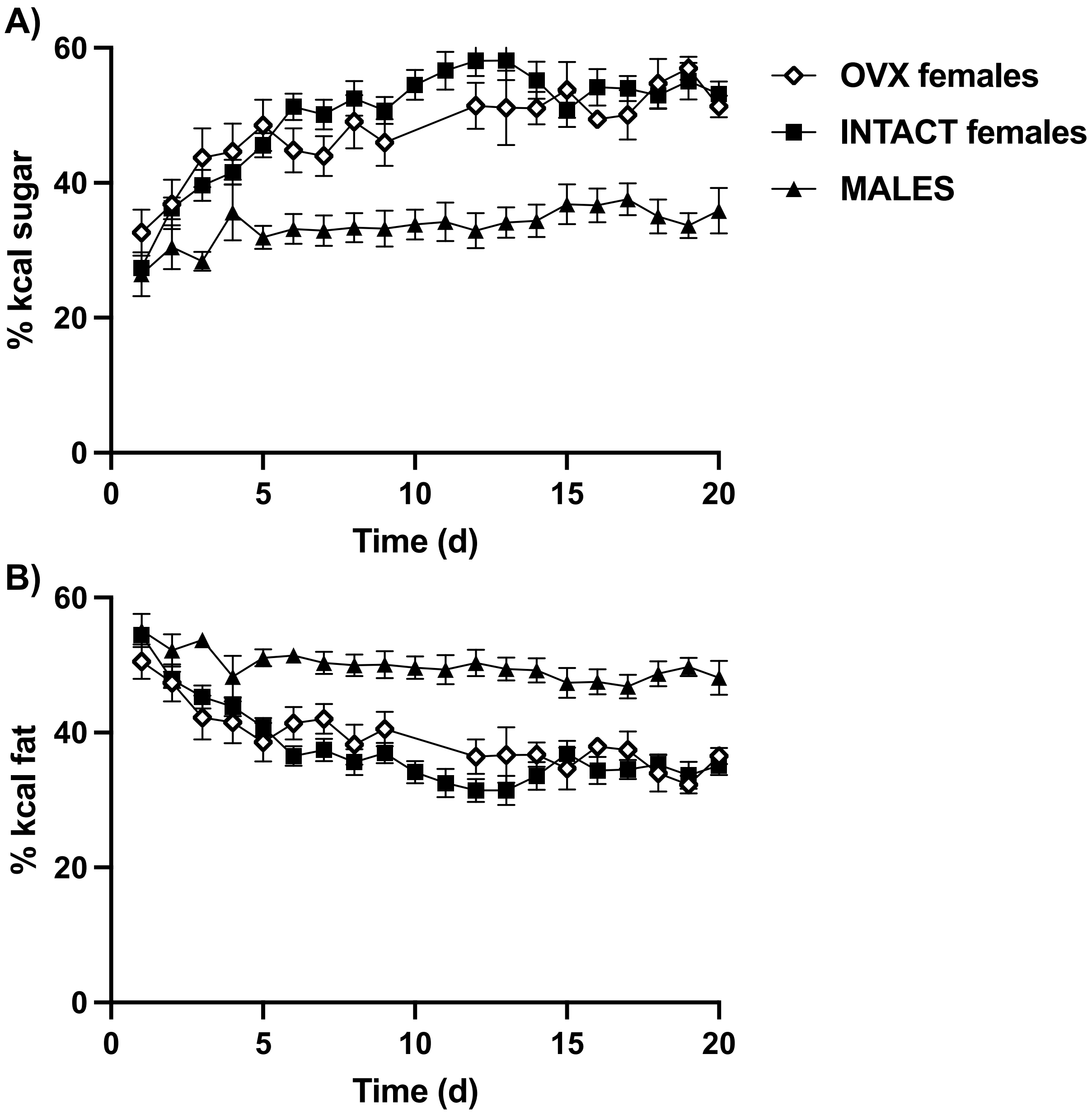

Supplemental Figure S3: Average intake and macronutrient preferences of ovariectomized females (n=5) on Western Diet containing 60% fat pellets and an 11% high fructose corn syrup. Ovariectomized (OVX) females display increased percentage of calories from sugar over time similar to non-ovariectomized (INTACT) females as compared to MALES (A). OVX and INTACT females decreased percentage of calories consumed from fat over time as compared to MALES (B). Data are shown as means ± SEMs.

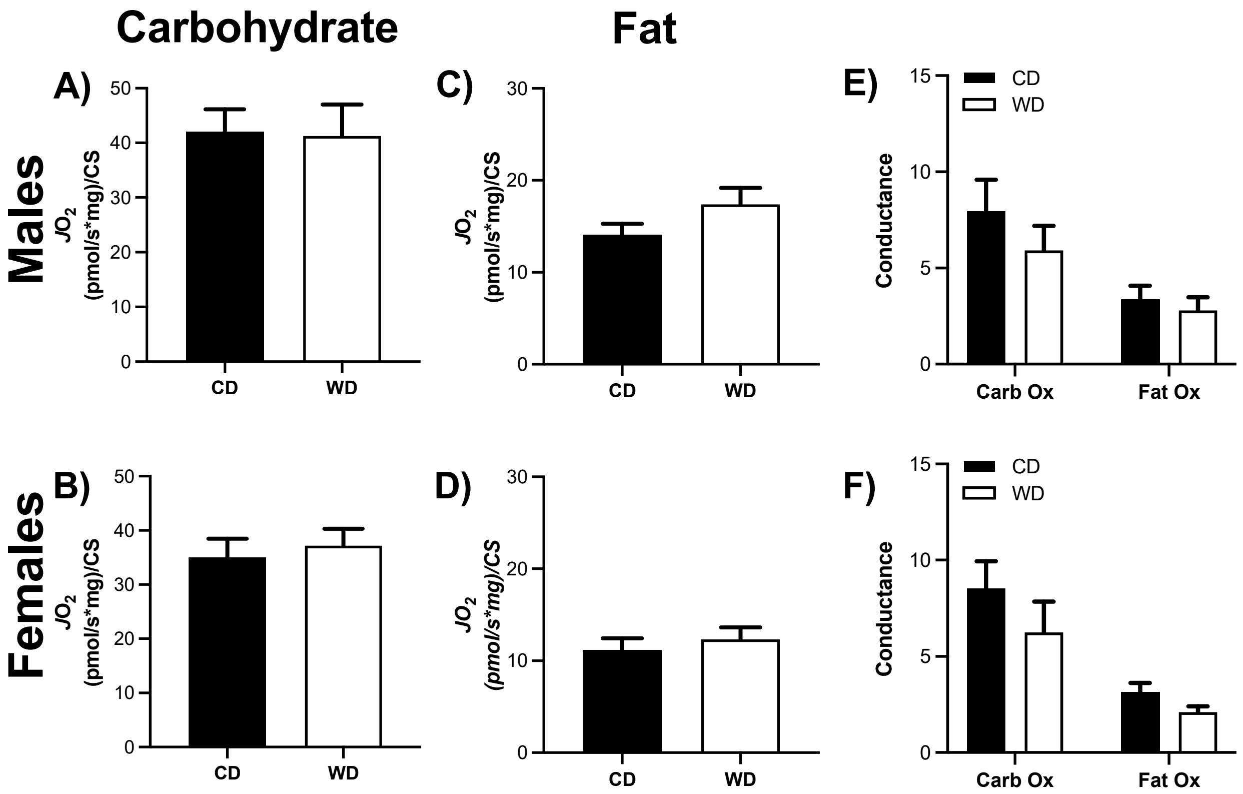

Supplemental Figure S4: Respiratory control in hippocampal mitochondria following consumption of a Western (WD) or control (CD) diet. C-D) Maximal mitochondrial respiration for carbohydrate substrates (A, B) and fat substrates (C, D) in male and female rats. Conductance slopes calculated from the linear phase of mitochondrial respiration (JO_2) as a function of ATP free energy (ΔG_{ATP}) for carbohydrate and fat substrates (E, F) in male and female rats. Data are analyzed using Student's two tailed t tests. Data are shown as means ± SEMs. *$P<0.05$.

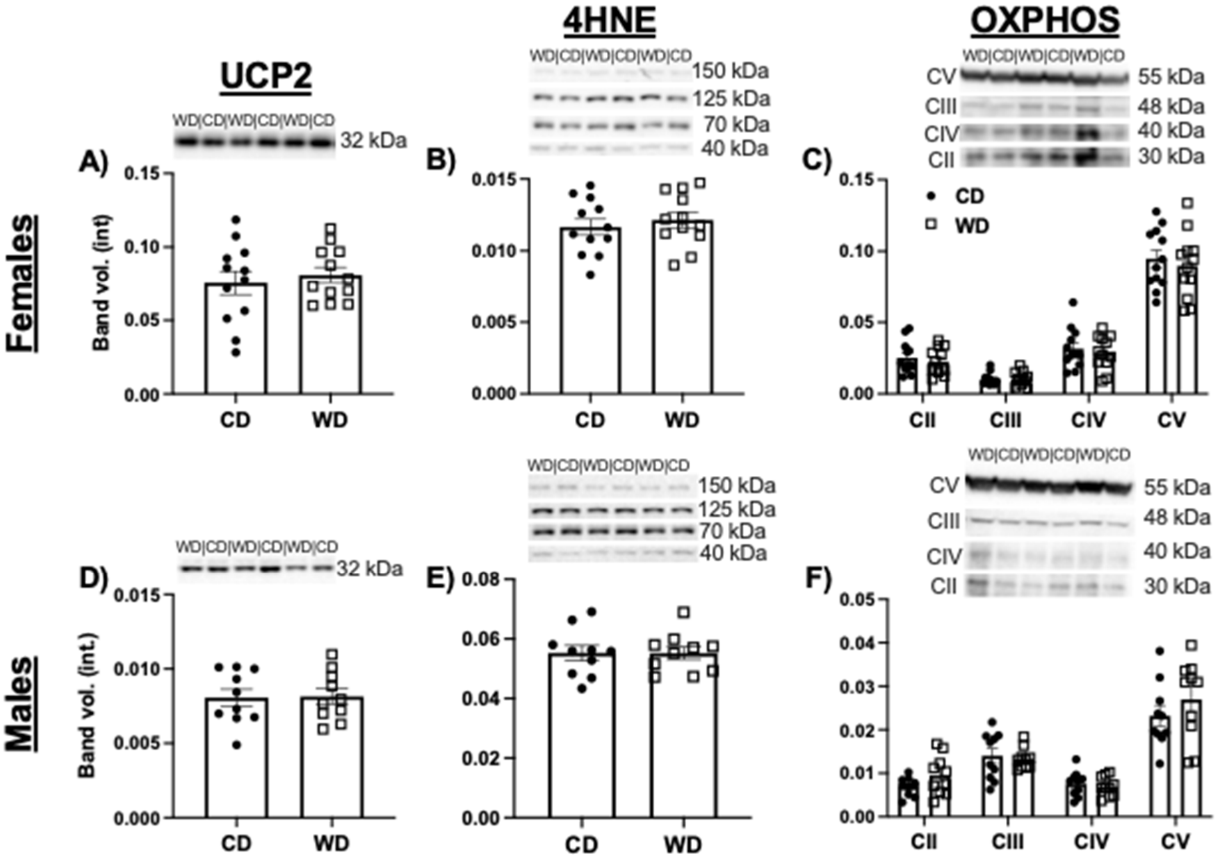

Supplemental Figure S5: Stain-free Western blotting in the hippocampus of rats fed either a Western (WD) or control (CD) diet. Levels of uncoupling protein-2 (UCP2) (A), 4-hydroxynonenol (4HNE) (B), and the oxidative phosphorylation (OXPHOS) complexes: succinate-Q oxidoreductase (CII), Q-cytochrome c oxidoreductase (CIII), cytochrome c oxidase (CIV), or ATP synthase (CV) (C) in female rats. Levels of UCP2 (D), 4HNE (E) and the oxidative phosphorylation complexes CII, CIII, CIV, or CV (F) in male rats. For all blots shown, lanes alternate treatment groups as indicated. All band intensities were normalized to total protein in the lane. Data are analyzed using Student’s two tailed t tests. Data are shown as means ± SEMs.

Supplemental Table S1: Citrate synthase activity

	Hippocampus		Hypothalamus	
	Male	Female	Male	Female
Control diet	145 (8)	187 (7)	180 (9)	176 (9)
Western diet	152 (9)	177 (8)	190 (12)	161 (11)
P-value	*0.568*	*0.357*	*0.513*	*0.303*

Supplemental Table 1: Values are mean +/- (SEM) µmol/min/g tissue wet weight. Control diet vs. Western Diet was analyzed by unpaired student's t-test.

Supplementary References

1. Santollo, J.; Eckel, L.A. Estradiol decreases the orexigenic effect of neuropeptide Y, but not agouti-related protein, in ovariectomized rats. *Behav Brain Res* **2008**, *191*, 173-177, doi:10.1016/j.bbr.2008.03.019.
2. Wood, R.I.; Rice, R. Ethanol-induced conditioned partner preference in female mice. *Behav Brain Res* **2013**, *243*, 273-277, doi:10.1016/j.bbr.2013.01.021.
